# The Armadillo Repeat Protein PF16 Is Essential for Flagellar Structure and Function in *Plasmodium* Male Gametes

**DOI:** 10.1371/journal.pone.0012901

**Published:** 2010-09-23

**Authors:** Ursula Straschil, Arthur M. Talman, David J. P. Ferguson, Karen A. Bunting, Zhengyao Xu, Elizabeth Bailes, Robert E. Sinden, Anthony A. Holder, Elizabeth F. Smith, Juliet C. Coates, Rita Tewari

**Affiliations:** 1 Institute of Genetics, School of Biology, University of Nottingham, Nottingham, United Kingdom; 2 Division of Cell and Molecular Biology, Imperial College London, London, United Kingdom; 3 Nuffield Department of Clinical Laboratory Science, University of Oxford, John Radcliffe Hospital, Oxford, United Kingdom; 4 Division of Parasitology, MRC National Institute for Medical Research, London, United Kingdom; 5 Department of Biological Sciences, Dartmouth College, Hanover, New Hampshire, United States of America; 6 School of Biosciences, University of Birmingham, Birmingham, United Kingdom; INSERM U1016, Institut Cochin, France

## Abstract

Malaria, caused by the apicomplexan parasite *Plasmodium*, threatens 40% of the world's population. Transmission between vertebrate and insect hosts depends on the sexual stages of the life-cycle. The male gamete of *Plasmodium* parasite is the only developmental stage that possesses a flagellum. Very little is known about the identity or function of proteins in the parasite's flagellar biology. Here, we characterise a *Plasmodium* PF16 homologue using reverse genetics in the mouse malaria parasite *Plasmodium berghei.* PF16 is a conserved Armadillo-repeat protein that regulates flagellar structure and motility in organisms as diverse as green algae and mice. We show that *P. berghei PF16* is expressed in the male gamete flagellum, where it plays a crucial role maintaining the correct microtubule structure in the central apparatus of the axoneme as studied by electron microscopy. Disruption of the *PF16* gene results in abnormal flagellar movement and reduced fertility, but does not lead to complete sterility, unlike *pf16* mutations in other organisms. Using homology modelling, bioinformatics analysis and complementation studies in *Chlamydomonas*, we show that some regions of the PF16 protein are highly conserved across all eukaryotes, whereas other regions may have species-specific functions. PF16 is the first ARM-repeat protein characterised in the malaria parasite genus *Plasmodium* and this study opens up a novel model for analysis of *Plasmodium* flagellar biology that may provide unique insights into an ancient organelle and suggest novel intervention strategies to control the malaria parasite.

## Introduction

Flagella and cilia are ancient cellular organelles used for motility, which are found in eukaryotic organisms ranging from unicellular protists to mammals. All flagella contain an axoneme, a structure consisting of a central apparatus (a pair of microtubules named C1 and C2) encircled by nine doublet microtubules. The axonemal microtubules have numerous associated structures including the central pair projections, radial spokes, and dynein arms. The dynein arms attached to the 9 doublet microtubules provide the force for generating motility. This “9+2” pattern of microtubules is conserved in most species and is thought to have been present in the common ancestor of all modern-day flagella and related structures (cilia) [1]. The complete axoneme is essential for well–regulated flagellar motility. Much of our knowledge of axoneme proteins and their mechanism of action has been obtained from studies on the green alga *Chlamydomonas* [2] and the protist *Trypanosoma* [3], [4], [5]. PF16 is an important Armadillo (ARM)-repeat protein of the central apparatus that was first functionally characterised in *Chlamydomonas* [6]. ARM-repeats consist of a ∼42-amino acid structurally conserved repeating motif named after the *Drosophila* segment polarity gene *Armadillo* (mammalian homologue, β-catenin) [7], [8]. Proteins containing ARM-repeats have diverse roles in eukaryotes, including cell signalling, cytoskeletal organisation and regulation of gene expression [9], [10]. Inactivation of the *Chlamydomonas PF16* gene led to loss of the central pair of microtubules and abnormal flagellar function, showing that PF16 is required for flagellar motility and stability of the central apparatus C1 microtubule [6]. Disruption of *SPAG6*, the mouse *PF16* orthologue, results in male infertility, due to loss of sperm motility [11]; and also to hydrocephalus, which is likely due to abnormal ciliary motility in epithelial cells in the brain cavities, as motile cilia are required to direct the correct flow of cerebrospinal fluid [12]. RNAi of *PF16* in the flagellated protozoan *Trypanosoma brucei* demonstrates a conserved role in flagellum-dependent motility [4], [13].

Malaria parasites belong to the genus *Plasmodium*, and are members of the phylum Apicomplexa. Malaria disease threatens 40% of the world's total human population. The human pathology of malaria is caused by parasite life-cycle stages that replicate asexually in red blood cells, but transmission to mosquitoes is achieved by the differentiation of the sexual stages: the male (micro-) and female (macro-) gametocytes that circulate within red blood cells in the vertebrate host in a developmentally arrested state. Gametocytes are activated to form gametes (a process known as gametogenesis) within the mosquito gut, following stimulation by environmental factors, including the drop in temperature from 37°C to 20–25°C and exposure to a mosquito metabolite, xanthurenic acid [14], [15]. Therefore, the sexual stages of *Plasmodium* are important potential targets for transmission-blocking strategies.

Microgametogenesis involves entry into the cell cycle, then three endomitotic divisions within 8 minutes, resulting in an 8-fold replication of the genome, followed by assembly of eight axonemes, which results in formation of eight motile uni-flagellated microgametes. Microgametes bud from the male gametocyte residing within the host's infected red blood cells within 10 to 15 minutes of activation, a “release” process known as exflagellation [14], [15]. The budding of male gametes requires flagellar beating, both to complete cytokinesis and to drive the axoneme and the membrane outwards from the residual gametocyte body. A male microgamete then fertilises a female macrogamete resulting in formation of a zygote that transforms into a motile ookinete [14], [15].

In malaria parasites the male gamete is the only stage in the life cycle with a flagellum. The structurally normal flagellum of *Plasmodium* differs in its method of formation from most other organisms as it is assembled within the cytoplasm of the microgametocyte and therefore does not rely on intra-flagellar transport (IFT) [15], [16], [17]. *Plasmodium* axonemes only become associated with the plasma membrane at the time of exflagellation, forming long thin motile gametes consisting almost entirely of flagellum [15]. Very little is known about the identity or function of proteins in the malaria parasite flagellum, although a specific tubulin gene is highly expressed in male gametes [18]. Here, we show using reverse genetics in a rodent malaria parasite, *Plasmodium berghei,* that an orthologue of PF16 (*Pb*PF16) plays a crucial role in male gamete flagellum biology and is involved in the assembly and/or stability of the flagellar central apparatus. We examine the consequences of *PbPF16* gene disruption for flagellar structure, movement and function and its effect on fertilization. We show that putative PF16 homologues have a wide phylogenetic distribution within eukaryotes. Homology modelling of *Plasmodium* and *Chlamydomonas* PF16 proteins highlights potential similarities and differences in their structure and function, which may explain why in our hands *Pb*PF16 is unable to complement the *Chlamydomonas pf16* mutant.

This study is the first molecular analysis of *Plasmodium* flagella and thus may provide unique insights into an ancient organelle that could identify novel intervention strategies to control the parasite.

## Results and Discussion

### PF16 gene deletion affects male gamete flagellar motility, male fertility and zygote formation

**Figure 1:**
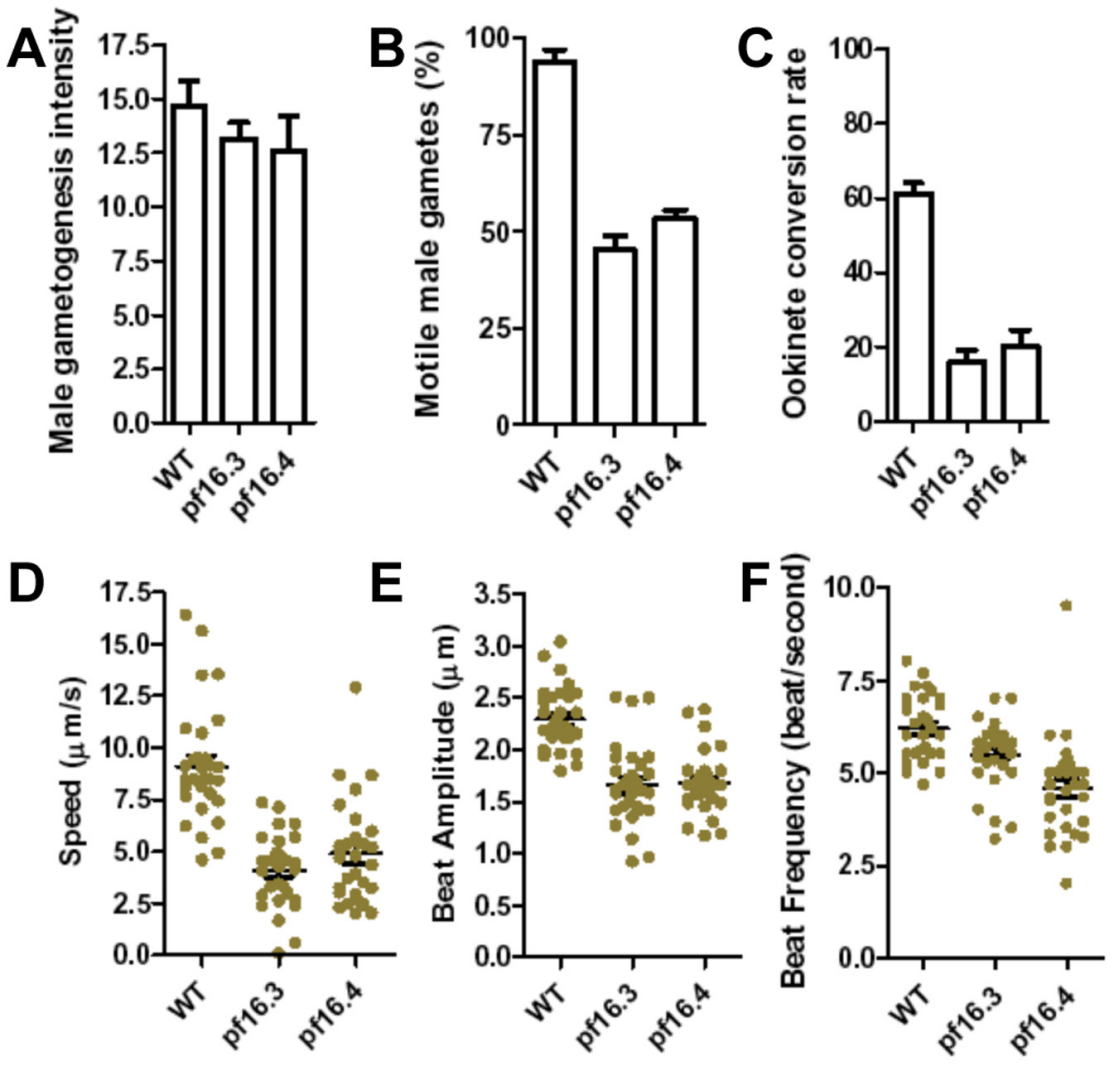
PF16 gene deletion reduces male gamete flagellar motility and ookinete (zygote) formation. A. Differentiation of male gametocytes to male gametes (as determined by quantifying exflagellation centres) is similar in both wild type *Plasmodium* (WT) and two *pf16* mutant clones (*pf16.3* and *pf16.4)* suggesting the emergence of male gametes is not affected (P = 0.3699 and P = 0.3789 for clones 3 and 4 respectively). Three independent replicates are plotted (established on 10 independent fields on a slide, with a 40x objective). B. Average number of motile gametes after emergence is reduced in both mutant clones (*pf16.3* and *pf16.4)* compared to wild type (WT) P = 0.0019 for both clones. Three independent replicates of 50 microgametes each were counted. C. Quantification of ookinete conversion (ratio of ookinetes to round cells expressed as a percentage). There was a decrease in the frequency of ookinete formation in both mutant clones when compared to wild type (WT). (P = 0.0017 and P = 0.0045 for clone 3 and 4 respectively). Three independent replicates of 100 events were counted (macrogametes + ookinetes). D, E, F. The male gamete flagellum was analysed for speed (D; P<0.0001 for both clones), beat amplitude (E; P<0.0001 for both clones) and beat frequency (F; P = 0.0180 and P<0.0001 for clones 3 and 4 respectively) in wild type and the two *pf16* mutant clones. In all these analyses the values were significantly lower (P<0.001) for both the mutant clones in comparison to wild type. See also Supplementary movie VS1and VS2. 30 male gametes from three independent samples were quantified for each analysis.

To determine the role of *P. berghei PF16* in parasite development we deleted the *PbPF16* gene (PB000781.01.0 or PBANKA_091740) by double homologous recombination (see supporting text). Note that the *PbPF16* gene is distinct from *Pfs16*, another gametocyte-specific gene. Two independent knockout clones, *pf16.3* and *pf16.4*, were generated in the *P. berghei* ANKA line “Figure S1” The growth of the asexual (blood) life-cycle stages of these two clones was unaffected; the rate of gametocyte formation was also similar to that of wild type parasites. The intensity of exflagellation (as a measure of male gametogenesis) was not altered in the *pf16* mutant lines compared to wild type parasite (Figure 1A), however these parasites displayed a severe defect in male gamete motility and fertility, with 50% of male gametes being immotile (Figure 1B,C). To investigate this defect further, we recorded flagellar beat frequency, amplitude and the speed of the male gamete flagella. The flagellar beat pattern of *pf16* mutant male gametes differed from wild type; it was characterised by a significantly lower beat frequency and beat amplitude together with a significantly reduced speed (Figure 1D,E,F). These defects led to a significant reduction in sexual fitness, i.e. the ability of male gametes from the *pbpf16* knockout lines to fertilise female(s) gametes (Figure 1), with a frequency of zygote (ookinete) formation of about 20%, as compared to 70 to 80% for wild type parasites (Figure 1C). These analyses clearly demonstrate a defect in the *pf16* male flagellum and its function (Figure 1 and “Videos S1 and S2”), which was reflected in the frequency of fertilization and zygote formation. The results are consistent with a function of PbPF16 in *Plasmodium* flagellar biology, similar to that in *Chlamydomonas* and mouse. However, deletion of *pf16* did not result in a total block in fertility, as observed in an *in vitro* ookinete assay here. This may be due to the relatively high abundance of female gametocytes and their proximity to male gametocytes in *Plasmodium* compared to other mammalian species [15]. It is also important to note that the zygotes (ookinetes) generated *in vivo* during a mosquito feed in the *pf16* mutant are able to give rise to a significant number of oocysts and generate normal sporozoites that are infectious to mice.

### Plasmodium PF16 is expressed in male gametocytes and gametes

**Figure 2:**
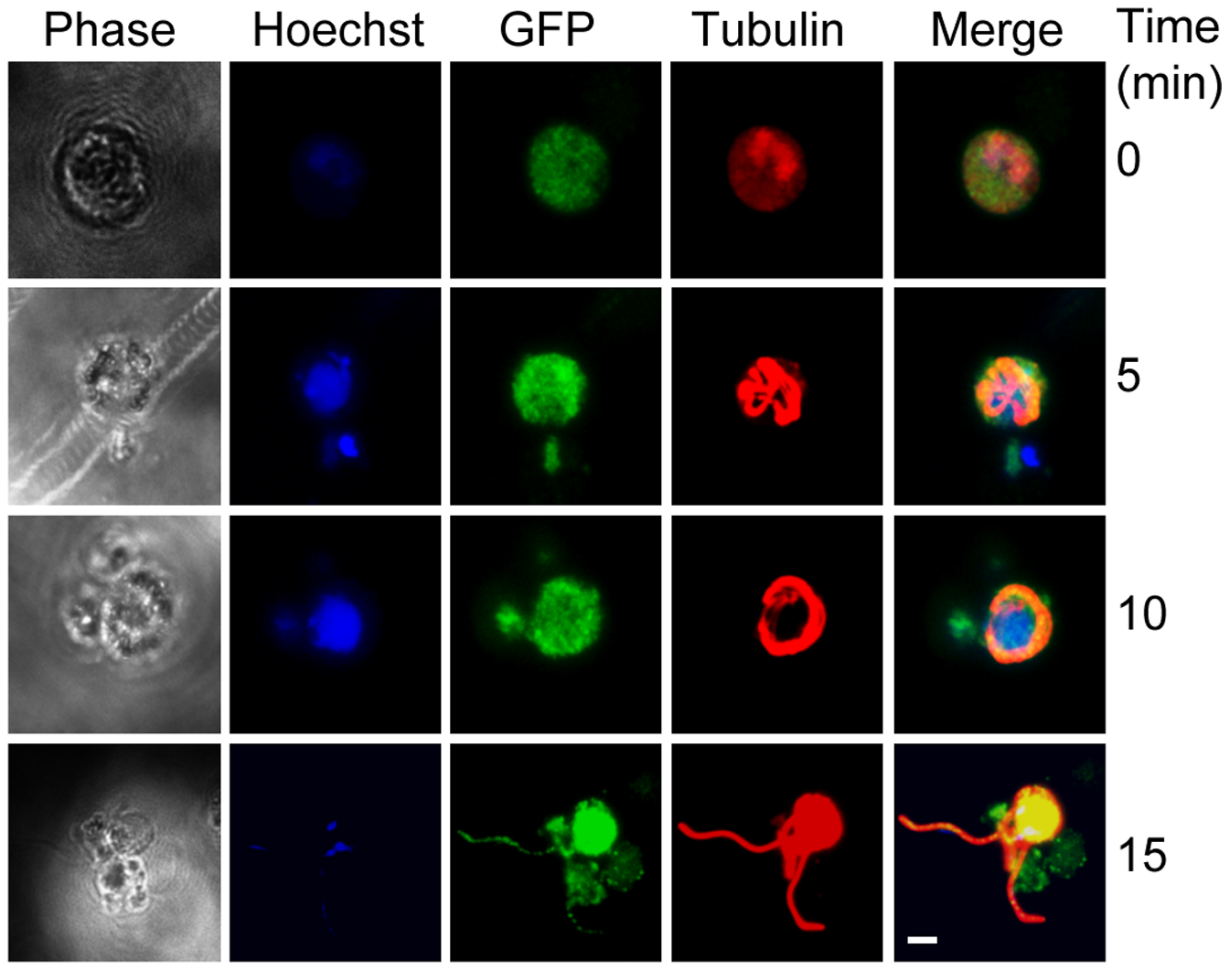
Plasmodium PF16 is expressed in the male gametocyte and gametes. Immunolocalisation of GFP in PF16-GFP-expressing parasites, both in the male gametocyte (0 min) and during male gamete formation during the exflagellation process (5–15 min). The parasites were co-stained with an anti tubulin antibody (red). Hoechst staining (blue) indicates the presence of DNA. Note the increase in the intensity of GFP localisation with the increase in time, and at the end of exflagellation when motile gametes form (15 min). Scale bar, 5 µm.

To examine the expression and localization of PbPF16 in the malaria parasite, we generated a transgenic “knock-in” *P. berghei* line expressing endogenous PbPF16 with a C-terminal green fluorescent protein (GFP) fusion “Figure S2”. These PbPF16-GFP transgenic parasites had no phenotypic abnormalities resulting from the expression of the PF16-GFP fusion protein. GFP expression was not visible in the asexual blood stage parasites, but faint GFP expression was detectable in the male gametocyte. Following gametocyte activation in exflagellation medium, we examined the location of PbPF16-GFP during gametogenesis. Before the onset of gametogenesis, PbPF16-GFP appeared as diffuse cytoplasmic fluorescence (Figure 2). At 5 and 10 minutes after activation, more intense PbPF16 was seen associated with growing cytoplasmic axonemes (detected by α-tubulin immunostaining; (Figure 2). Later, when male gametes were emerging from the residual body of the male gametocyte, PbPF16-GFP was clearly localised to the emerging male gamete and had patchy distribution along the length of the gamete; moreover it appeared that PbPF16-GFP was always encompassed within the axonemal fluorescence (Figure 2). The protein was not detected in activated female gametes or in the ookinete at either 4 hours or 24 hours after activation. These results clearly show that *PbPF16* is expressed as a male gamete-specific gene and that the protein is expressed at a higher level (or in a more visible form) in the activated male gametocyte/gamete.

### Plasmodium PF16 affects the formation of the central apparatus of the axoneme in male gamete flagella

**Figure 3:**
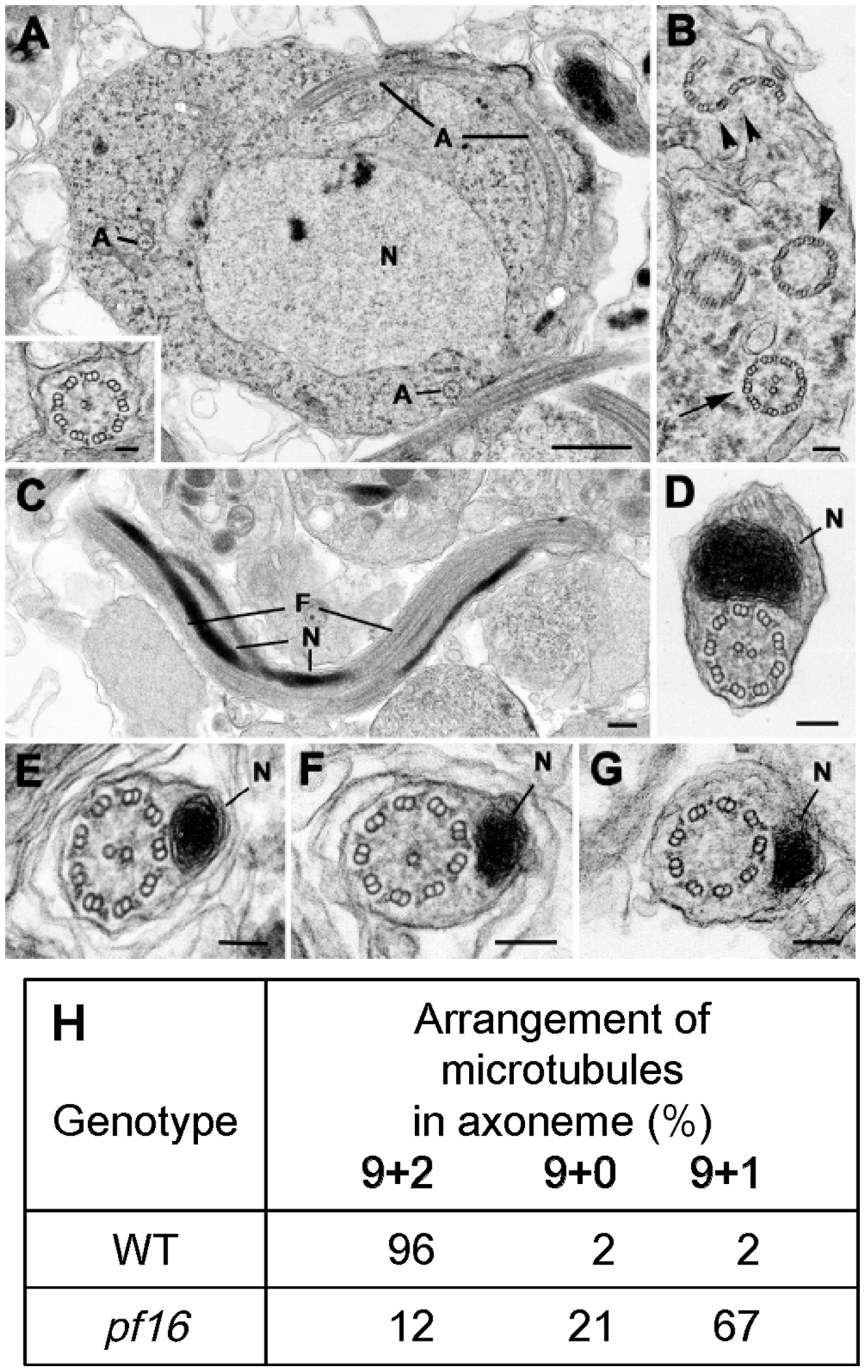
Comparison of the microgametocytes and microgametes of wild type P. berghei and pf16 mutant P. berghei by electron microscopy. A. Section through a wild type microgametocyte showing the large central nucleus (N) and longitudinal- and cross-sections of axonemes (A) within the cytoplasm. Scale bar, 1 µm. **Insert.** Enlargement of an axoneme cross-section from a mutant microgametocyte showing the presence of a single central microtubule (9+1). Scale bar, 100 nm. B. Detail of the cytoplasm of a wild type microgamete illustrating the variability of the axoneme structure: some have two central microtubules (arrow), others have no central tubules (arrowhead), some peripheral duplet microtubules form an “S” shaped structure (double arrowheads). Scale bar, 100 nm. C. Longitudinal section through a wild type microgamete showing the undulating axoneme forming the flagellum (F) with the closely adhering electron-dense nucleus (N). Scale bar, 100 nm. D. Cross-section through the central region of a wild type microgamete showing the nucleus and the normal 9+2 organisation of the axoneme. Scale bar, 100 nm. E-G. Cross-sections through microgametes of the PF16 mutant showing the variable axonemal appearance with a few showing the normal 9+2 structure (E), the majority showing 9+1 (F) and a number with a 9+0 appearance (G). Scale bar, 100 nm. H. Quantification of microtubule arrangements in the axonemes of wild type (WT) and *pf16* mutant microgametes.

To determine any possible defect in flagellar morphology, the ultrastructure of the axoneme and flagellum was examined in wild type and *pf16* mutant parasites (Figure 3A; [15]). The development of the axonemes is unusual, as they do not arise from a classical centriole/basal body with nine concentric triple microtubules. Instead, there is an electron dense structure in which nine single microtubules have been identified [15]. This can result a slightly uncoordinated assembly process where the majority of axonemes consist of a central pair of microtubules (central apparatus) encircled by 9 doublets of microtubules with associated dynein arms (Figure 3B; [15]). However, some axonemes lack the central pair (9+0) or the peripheral doublets forming an “S” shape rather than a circle (Figure 3B). Similar structures were observed in the *pf16* mutant, however a significant number of the axonemes have one missing central tubule (9+1) (Figure 3A insert; see below). The mature microgamete consists of an elongated, undulating axoneme with a flattened electron-dense nucleus in the central region, which follows the contours of the axoneme, all enclosed by a unit membrane (Figure 3C). In cross sections of wild type parasites, the vast majority (96%) displayed the typical 9+2 arrangement with only rare examples of 9+0 (2%) or 9+1 (2%) (Figure 3D,H). However, the *pf16* mutant clones showed a marked increase in the number of microgametes with atypical axonemes (Figure 3). Only 12% had the normal 9+2 structure (Figure 3E,H) while 21% had 9+0 (Figure 3F,H) and 67% had a 9+1 structure (Figure 3G,H). Therefore, the loss of *PbPF16* results in increased numbers of abnormal microgametes with the majority lacking one central microtubule (9+1) and a significant number lacking both central microtubules (9+0) (Figure 3E–H).

This observation is consistent with an important role of PbPF16 in axoneme formation in *Plasmodium*, with its absence affecting formation of the central apparatus. The result suggests that PF16's function in promoting the stability/assembly of a single microtubule of the central apparatus is conserved between *Plasmodium* and *Chlamydomonas* despite their evolutionary divergence and differences in the way flagella are formed in the two species. It is interesting to note that in *Chlamydomonas*, mutations in *pf16* result in the failure of assembly of three axonemal proteins including PF16; however, upon demembranation of the flagella, the C1 microtubule is unstable and disassembles [19]. This phenotype is similar to that in *pf16* mutants in other organisms such as mouse or Trypanosomes where only 20–30% of mutant flagella exhibit the loss of the central microtubules [4], [11]. In contrast, our results show that in *Plasmodium*, almost 90% of flagella lack one or two microtubules of the central pair in the absence of detergent to remove the flagellar membrane. This result may be explained by differential defects in the assembly of these microtubules. In organisms such as *Chlamydomonas*, PF16 appears to only affect the assembly of two other central apparatus-associated proteins, which in turn, affect the stability of the C1 central microtubule. In other organisms such as *Plasmodium*, PF16 may play a more significant role in assembly of the central microtubules. Additional roles for PF16 in flagellar assembly are suggested by the observation that PF16 localises to both the basal body and the central apparatus in the ciliate *Tetrahymena*, and undergoes dynamic exchange in the basal body [20], [21].

### Complementation studies with Plasmodium PF16 in Chlamydomonas

To test whether or not the sequence similarity between the *P. berghei* PF16 protein and *Chlamydomonas* PF16 would confer functional similarity, we transformed a *Chlamydomonas pf16* mutant with the complete *P. berghei PF16* cDNA using either the *P. berghei* or *Chlamydomonas* 5′ and 3′ UTRs. For each experiment, the 5′ UTR contained either the *P. berghei* PF16 promoter or the *Chlamydomonas* PF16 promoter (supporting material text). No rescue of the motility defect was detected in over 700 transformants from three independent experiments.

There are several explanations for the lack of complementation. It is possible that *Chlamydomonas* was unable to efficiently express the *Plasmodium* gene. The *Chlamydomonas* genome is highly GC-rich, whereas the *Plasmodium* genome is AT-rich, which may affect translation. One possibility for future experiemtns we would be to use a codon-optimised *P. berghei PF16* gene. Additional regulatory elements may also be required for successful gene expression in *Chlamydomonas;* for example, expression of foreign genes in *Chlamydomonas* may be significantly improved by the introduction of *Chlamydomonas* introns that possess enhancer elements [22]. Finally, it is possible that the *Plasmodium* protein is unable to interact with additional *Chlamydomonas* components required for assembly into the axoneme. This hypothesis was explored using homology modelling.

### Homology model structure analyses of Plasmodium berghei PF16

**Figure 4:**
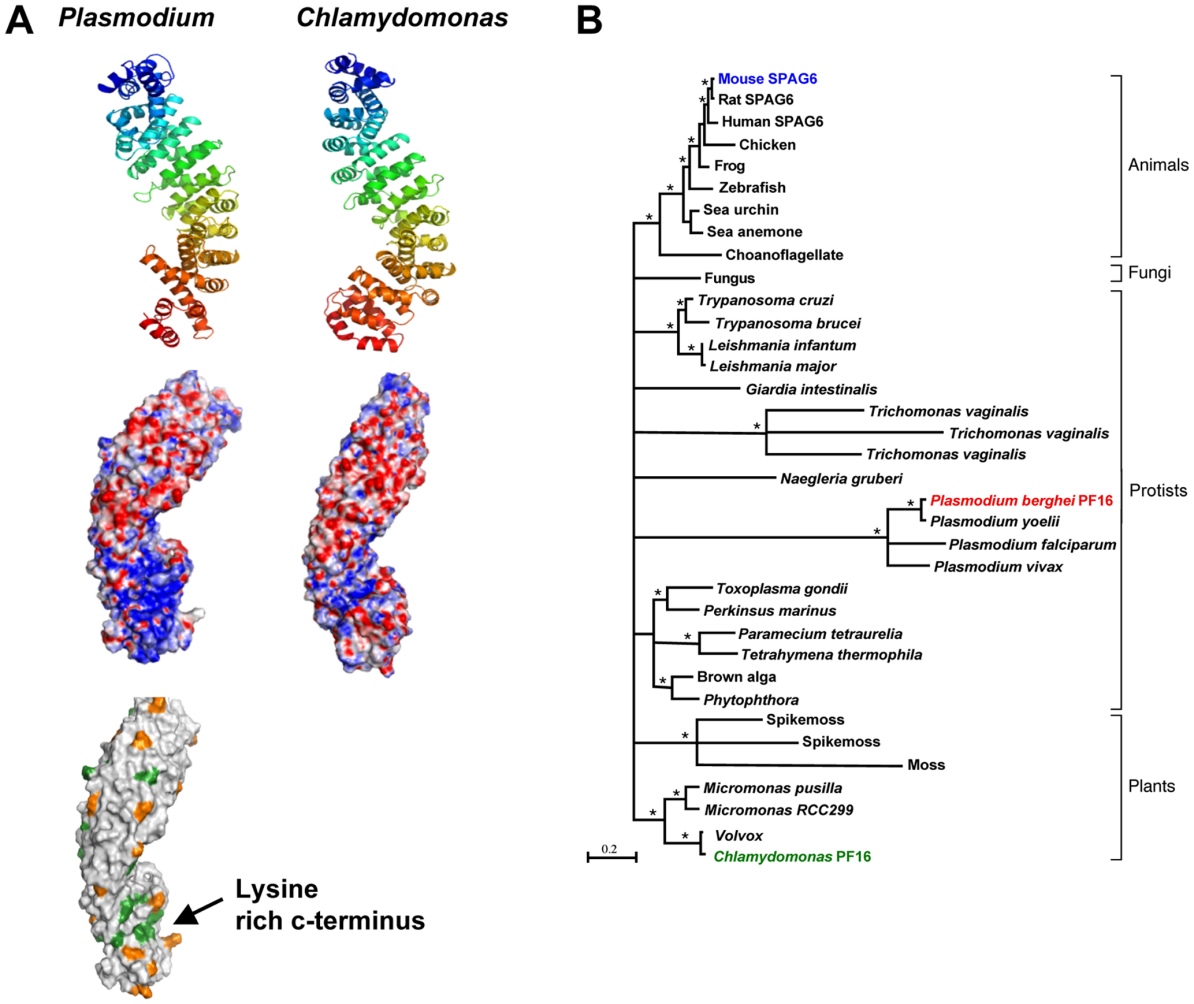
Comparison of Structure and Sequence analyses of Plasmodium berghei PF16. A. Top view – cartoon representation of homology models of *Plasmodium* and *Chlamydomonas* PF16, coloured by spectrum from N-terminus (blue) to C-terminus (red). Middle view – electrostatic surfaces of the modelled proteins (view rotated 180° around the *y* axis with respect to top view). The accessible surface area is coloured according to electrostatic potential calculated using APBS from -10k_B_T/e (red) to + 10k_B_T/e (blue) [47]. Bottom view – surface representation of *Plasmodium* PF16 displaying exposed lysine residues. Those shown in green represent lysine residues conserved in *Chlamydomonas* PF16, with those in orange representing lysine residues found only in *Plasmodium* PF16. Figures produced using PyMol (The PyMOL Molecular Graphics System, Version 1.2r3pre, Schrödinger, LLC). B. Maximum likelihood tree of proteins from 33 different species. Mouse SPAG6 (blue), *P. berghei* PF16 (red) and *Chlamydomonas* PF16 (green) are highlighted. The accession number of each species is given in the supplementary material. The tree was made using the WAG model of protein evolution with gamma distributed rates at sites and 1000 bootstrapped replicates implemented in PhyML [48]. Branches with bootstrap support less than 70% are collapsed. Asterisk (*) signifies bootstrap support greater than 90%. The scale bar indicates 0.2 substitutions per site.

In order to compare the structures of PbPF16 and *Chlamydomonas* PF16, both protein structures were determined using homology modeling. Protein sequences were modeled using the I-TASSER server, using robust alignment procedures followed by simulation and refinement of subsequent models [46]. The models for the two sequences clearly demonstrate the characteristic architecture of ARM-repeat proteins [7], [9]; Figure 4A) despite the fact that *Plasmodium berghei PF16* is AT-rich (24% GC) and the amino acid sequence identity between *Pb*PF16 and *Chlamydomonas PF16* is low (37%; Figure 4B). In each protein, nine tandem ARM-repeats pack together to form a right-handed superhelix (Figure 4A; “Figure S3”.) Root mean square differences ranging from 0.88 to 3.26 Å indicate structural conservation between the target and template structures. Protein-protein interactions are crucial to the cellular function of ARM-repeat proteins [7], [9]. We therefore compared the properties of the two structures to identify differences in surface exposed residues. The *Plasmodium* PF16 modelled sequence has a significantly higher theoretical pI (8.1) compared to either *Chlamydomonas* PF16 (5.69) or human SPAG6 (6.51), largely attributable to a significant increase in lysine (11.5% of residues) compared with *Chlamydomonas* (5.3%). We calculated the surface electrostatic potential for the models of *P. berghei* and *Chlamydomonas* PF16s, and this indicated a basic patch in the C-terminal region of *P. berghei* PF16 not present in *Chlamydomonas* (Figure 4A). Residues contributing to this patch are conserved in all the *Plasmodium* species (*P. falciparum*, *P. vivax*, *P. knowlesi*, *P. chaubaudi*, *P. yoelii*, *P. berghei).* Other species (*Toxoplasma*, mouse and human) PF16s retain a number of the positively charged residues involved in this region and show an intermediate basic character between the *Plasmodium* species and *Chlamydomonas* PF16s. A small number of lysine residues are conserved in all species. The basic nature of *P. berghei* PF16 may be refractory to at least some of the protein-protein interactions required for the function of *Chlamydomonas* PF16, thereby preventing the *P. berghei* orthologue from functioning in *Chlamydomonas*.

### Bioinformatic analyses of putative PF16 homologues in Apicomplexan parasites and other eukaryotic genomes

We determined how *Plasmodium* PF16s relate more widely to other eukaryotic PF16s. Firstly, putative PF16 protein sequences from different species were identified, aligned and a tree constructed (Figure 4B).

Within the phylum Apicomplexa, there are numerous parasites causing disease in humans and domestic animals, including the genera *Theileria, Babesia*, *Cryptosporidium, Plasmodium, Toxoplasma* and *Eimeria*. In the latter three genera there is a distinctive sexual phase resulting in the production of sperm-like motile microgametes and egg-like immotile macrogametes. The motility of these microgametes is based on the presence of flagella; two in *Toxoplasma* and *Eimeria* and one in *Plasmodium* [16], [17], [23]. In *Toxoplasma* and *Eimeria* flagella formation occurs by the axoneme budding from the surface in a similar manner to most other organisms. In contrast, *Plasmodium* species' flagellum only becomes associated with the plasma membrane at the time of release as described earlier. Putative PF16 homologues are present in *Plasmodium*, *Toxoplasma* and *Eimeria* (Figure 4B; [16], [17]). A PF16 homologue appears to be absent from *Babesia*, *Cryptosporidium* and *Thelieria*, which lack flagella. However, in *Babesia*, ray bodies, which resemble microgametes but lack an axoneme are present [24], [25]. Thus in the Apicomplexa there is an association between the presence of the PF16 homologue and flagellated gametes.

PF16 homologues are found in non-apicomplexan chromoalveolates (Figure 4B), including flagellated plant-pathogenic oomycetes of the *Phytophthora* species, and the ciliated protists *Paramecium* and *Tetrahymena*. The brown alga *Aureococcus* also has a putative PF16 homologue but no known flagellum: *Aureococcus* may have an undiscovered flagellated stage in its life cycle, due also to the unexpected presence of other flagellar proteins [26]. Curiously, PF16 homologues are apparently absent from flagellated diatoms, which lack the central pair of microtubules ([27]; Figure 4B).

A PF16 homologue is present in the Trypanosomatids, where it functions in the life cycle stages that possess a motile flagellum [4], [28]. We also find putative PF16 homologues in other flagellated excavates, including the parasites *Leishmania* and *Trichomonas*, and the non-pathogenic *Naegleria gruberi*, which has both flagellated and amoeboid forms (Figure 4B). In *Trichomonas vaginalis* there are three proteins divergent from proteins in other excavates and from each other. *T. vaginalis* is reported to have undergone recent genome duplication [29].

PF16 homologues were not detected in higher plants, which lack flagella; however the moss *Physcomitrella* and the lycophyte (spikemoss) *Selaginella*, which have flagellated male gametes, possess putative PF16 homologues, as do a variety of flagellated green algae including *Chlamydomonas* (Figure 4B). PF16 homologues also appear to be present in the flagellated fungus *Batrachochytrium*, and in the Choanoflagellate, *Monosiga brevicollis*. Thus, it appears that PF16 homologues are found throughout flagellated eukaryotes. However, it is interesting to note that PF16 is absent from *C. elegans*, which possesses only sensory (non motile) cilia [16].

### Plasmodium PF16: a starting point for understanding Plasmodium flagellar biology?

In this study we demonstrate that the ARM-repeat protein, *P. berghei* PF16, identified by similarity with *Chlamydomonas* PF16, is important for male gamete flagellum biology in the malaria parasite. Thus although the mechanism of flagellar assembly differs between the two systems [15], PF16 has retained a conserved function in regulating the pair of microtubules in the central apparatus of the axoneme. In our earlier studies we have shown that certain *Plasmodium*-specific kinases control exflagellation, tubulin polymerisation and cytokinesis during male gametogenesis [30], [31]. In light of these studies it will be interesting to examine how PbPF16 relates to phosphorylation pathways that control the process of flagellum formation.

Cilia and flagella are ancient organelles and have been widely studied. They have been linked to number of human genetic diseases, primarily in ciliary dyskinesias, abnormal epithelial cilia and sperm flagella (for a review see [32], [33]). What controls the movement of cilia and flagella has been an interesting point of study in theoretical physics [34]. Among unicellular protists, elegant studies in *Trypanosoma* have dissected the function of the flagellum and shown that it is a multifunctional organelle, having a role at various stages during the life cycle of the parasite [3], [4], [5]. This contrasts with *Plasmodium*, where flagella are formed only during male gametogenesis and are therefore essential only for fertilisation and transmission. During our search for other components of the central pair apparatus we found that there are many other putative proteins in *Plasmodium* that are likely to interact with PF16 and regulate the assembly of the axoneme as in *Chlamydomonas* and other eukaryotes [4], [27], [35]. For example, *Plasmodium* has a putative homologue of PF20, an important component of the central apparatus that interacts with both PF16 and Fused kinase [27]. However, Fused kinase appears to be absent from *Plasmodium* even though *fused* genes have been found in *Leishmania* and *Trypanosoma* [27]. Our study in *Plasmodium* raises various questions as to how the central apparatus is organised during the formation of the flagellum in the malaria parasite. It will be crucial to identify the partners interacting with *Plasmodium* PF16. Investigating the role and function of various proteins in *Plasmodium* flagellar biology will provide a unique way to understand this ancient organelle, as well as the sexual differentiation of male gametocytes, and hence design novel intervention strategies to prevent the transmission of malaria.

## Materials and Methods

For targeting and tagging constructs and genotype analyses of transgenic parasite please see “Materials and Methods S1”.

### Ethics statement

All animal work has passed an ethical review process and was approved by the United Kingdom Home Office. Work was carried out in accordance with the United Kingdom “Animals (Scientific Procedures) Act 1986” and in compliance with “European Directive 86/609/EEC” for the protection of animals used for experimental purposes. The permit number for the project licence is 40/3344.

### Exflagellation and male gamete motility assays

Mice were treated intraperitoneally (i.p.) with 0.2 ml of 6 mg/ml phenylhydrazine (BDH Chemicals Ltd, UK) to induce hyper-reticulocytosis two to three days prior to infection. Parasites were harvested by heart-puncture at day 3 or 4 post-infection (p.i.). On day three post-infection, tail blood was harvested and resuspended in 1 volume of exflagellation medium (RPMI 1640 (Sigma, UK) containing 100 µM xanthurenic acid (Sigma, UK), pH 7.4). For the exflagellation assay, the number of exflagellation events was recorded 14 minutes after activation, on 10 fields. For male gamete motility assays, the suspension of blood was spun down at 500 g 15 minutes after activation; the supernatant containing male gametes was then harvested and mounted on a slide. Male gametes were visualized by phase contrast microscopy on a Leica DMR microscope and 5-second videos were captured with the Zeiss AxioCam HRC camera and Axiovision software. Videos were taken at a speed of 16 images per second. Individual gametes were tracked one at a time and analyses of beat frequency, amplitude and speed were recorded manually from individual video frames.

### Ookinete conversion assay

Parasite-infected mosquito blood was resuspended in ookinete medium as described earlier [31], [36]. After 24 hours, the samples were resuspended and added at a 1∶1 ratio to Cy3-conjugated 13.1 antibody (13.1 is an antibody specific to macrogametes and ookinetes [31], [37] The proportion of ookinetes to all 13.1-positive cells (unfertilised macrogametes and ookinetes) was established.

### Immunocytochemistry

Parasite-infected blood (as described above) was resuspended in 4% paraformaldehyde (PFA) (Novagen, UK). Immunocytochemistry was performed on the fixed parasite material using the primary antibodies rabbit anti-GFP (ABCAM, USA, used at 1 in 200dilution) and mouse monoclonal anti-alpha tubulin (Sigma, UK, used at 1 in 500). Secondary antibodies used were Alexa 488-conjugated anti-rabbit IgG and Alexa 547 conjugated anti-mouse IgG (Molecular probes, UK, both used at 1 in 1000). The slides were then mounted in Vectashield with DAPI (Vector Labs). Parasites were visualized on a Leica SP5 confocal microscope and data acquired and analysed with the LAS AF Lite software (Leica, UK).

### Statistical analyses

All statistical analyses were conducted with GraphPad Prism (GraphPad Software, USA). Non-parametric t-tests were used to compare exflagellation intensities, conversion rates and male motility patterns.

### Electron microscopy

Samples of wild type and *pf16* microgametocytes and microgametes cultured as described above were fixed in 4% glutaraldehyde in 0.1M phosphate buffer and processed for routine electron microscopy as described previously [38]. In summary, samples were post fixed in osmium tetroxide, treated en bloc with uranyl acetate, dehydrated and embedded in Spurr's epoxy resin. Thin sections were stained with uranyl acetate and lead citrate prior to examination in a JEOL12EX electron microscope. To quantify structural differences in the axonemal appearance, a random sample of 100 cross-sectioned microgametes was examined.

### Complementation studies in Chlamydomonas

For *Chlamydomonas* rescue experiments *pf16C,arg-* cells [6], [39] were co-transformed with the plasmid of interest with either *P. berghei* coding sequence with its control element or containing *Chlamydomonas* control elements as well as the pArg7.8 plasmid containing a selectable marker (arginino-succinate lyase gene [40]. The details of plasmid construct are in the supplementary material text “Method S”.

### Molecular sequence analyses

Similarity searches were made using BLASTP [41] against NCBI databases and genome sequence protein databases [42], [43]; www.jgi.doe.gov/genome-projects/). Putative homologues were aligned using ClustalW [44] and minor manual adjustments were made using SEAVIEW [45].

### Homology modelling

Protein structure prediction was performed using the I-TASSER server, submitting sequences for *P. berghei* PF16, human PF16 (SPAG6) and *Chlamydomonas* PF16 (residues 1–463, 1–510 and 1–476 respectively). I-TASSER undertakes threading, utilising profile-profile alignment, followed by structure assembly simulation and refinement [46].

## Supporting Information

Figure S1 Targeted disruption of PF16 gene in *P. berghei.* A. Schematic representation of gene targeting construct used for gene replacement by double homologous recombination. Position of primers 1–4 is (used for diagnostic PCR) is indicated, along with the restriction enzyme site EcoRI used for Southern hybridisation. B. Diagnostic PCR verifying disruption of *PF16* locus in mutant clones *pf16.3 (16.3)* and *pf16.4 (16.4)*. Primer set 1/2 was used to detect the unique product across the integration site and primer set 3/4 was used to test the absence of *PF16* gene. C. Southern hybridisation of EcoRI-digested DNA using the 3′UTR from targeting construct as probe. Arrow indicates the diagnostic bands for the mutant clones *pf16.3* and *pf16.4* (*16.3* and *16.4* respectively). D. Pulse field gel electrophoresis blot hybridised with *P. berghei* 3′UTR that detects the endogenous locus at chromosome 7 and the disrupted locus at Chromosome 10 in clone 4 (*16.4*) (arrow).
(0.47 MB TIF)

Figure S2 GFP Tagging of endogenous PF16 in *P. berghei.* A. Schematic representation of gene tagging construct used for endogenous GFP tagging by single homologous recombination. Position of primers 1–4 is (used for diagnostic PCR) is indicated B. Diagnostic PCR for integration of the transgenic construct for generating endogenous PF16-GFP parasites. Primer 1/2 shows integration across the region and 3/4 detects the template control. W represents the wild type and transgenic PF16-GFP is represented by (T) C. Correct integration of the transgenic construct (T) at chromosome 10 as described above, compared to the wild type locus (W). D. Western blot analyses using an anti-GFP antibody on control wild type gametocytes/gametes ubiquitously expressing soluble GFP (W) and transgenic male PF16-GFP-expressing gametes (T).
(0.47 MB TIF)

Figure S3 Structural Models of PF16. Comparison of the modelled *Plasmodium*, human and *Chlamydomonas* PF16 proteins with experimentally determined structures demonstrating the conserved nature of the ARM repeat. Structures shown are human β-catenin [PDB:1JDH], mouse importin α [PDB:1IAL] and yeast karyopherin α [PDB:1EE4]. All structures are shown in cartoon representation, coloured by spectrum from the N-terminus (blue) to C-terminus (red).
(0.97 MB TIF)

Video S1 Wild type male gamete flagella movement
(2.89 MB MOV)

Video S2 pf16knockout male gamete flagella movement
(3.31 MB MOV)

Materials and Methods S1 (0.06 MB DOC)

References S1 (0.02 MB DOC)
