# Supplementary figures and images for: The Armadillo Repeat Protein PF16 Is Essential for Flagellar Structure and Function in Plasmodium Male Gametes
Source: PLoS One. 2010 Sep 23;5(9):e12901. doi: 10.1371/journal.pone.0012901 (PMC2944832; doi:10.1371/journal.pone.0012901)

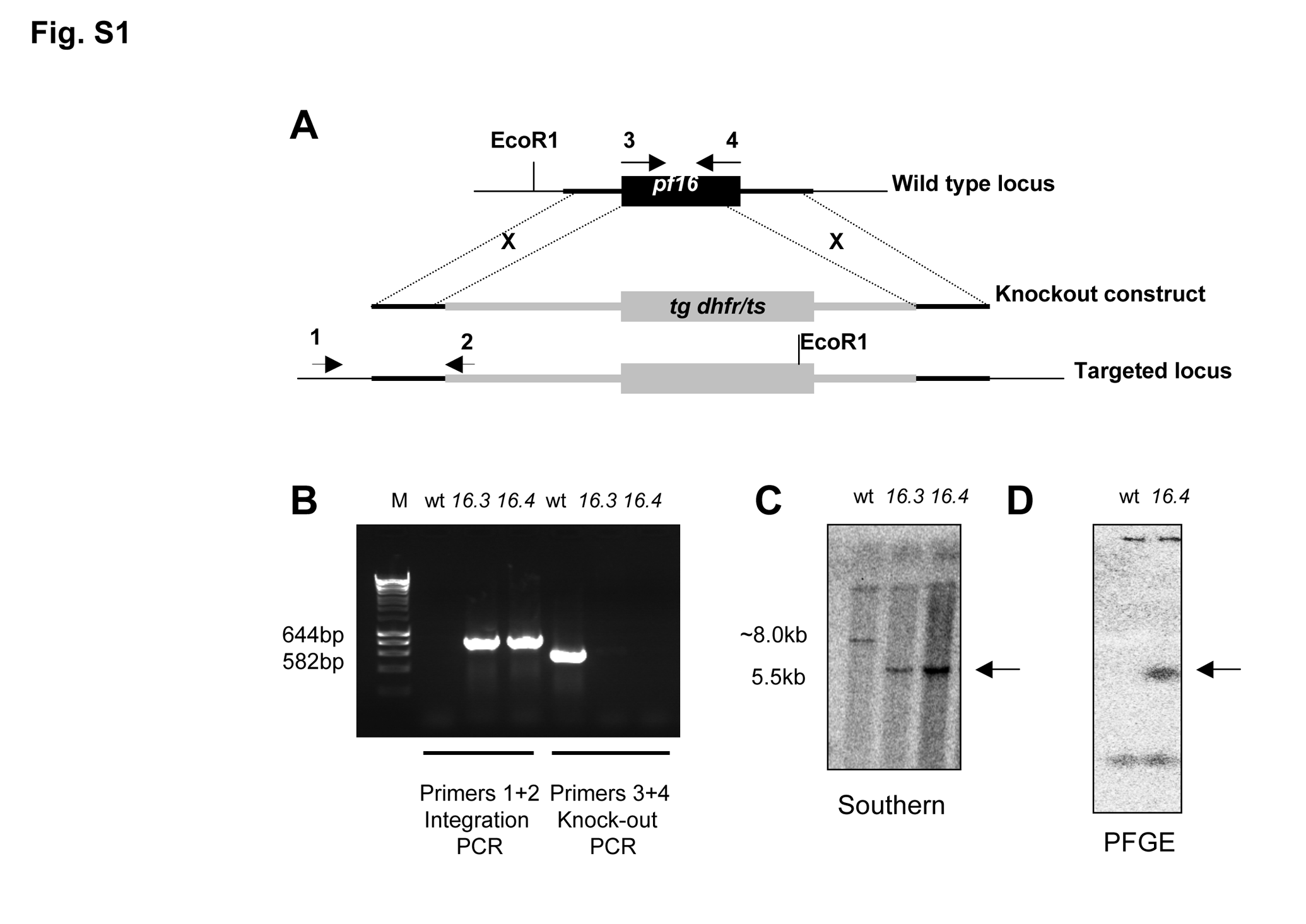

Supplement: Figure S1 — Targeted disruption of PF16 gene in P. berghei. A. Schematic representation of gene targeting construct used for gene replacement by double homologous recombination. Position of primers 1–4 is (used for diagnostic PCR) is indicated, along with the restriction enzyme site EcoRI used for Southern hybridisation. B. Diagnostic PCR verifying disruption of PF16 locus in mutant clones pf16.3 (16.3) and pf16.4 (16.4). Primer set 1/2 was used to detect the unique product across the integration site and primer set 3/4 was used to test the absence of PF16 gene. C. Southern hybridisation of EcoRI-digested DNA using the 3′UTR from targeting construct as probe. Arrow indicates the diagnostic bands for the mutant clones pf16.3 and pf16.4 (16.3 and 16.4 respectively). D. Pulse field gel electrophoresis blot hybridised with P. berghei 3′UTR that detects the endogenous locus at chromosome 7 and the disrupted locus at Chromosome 10 in clone 4 (16.4) (arrow). (0.47 MB TIF) [file pone.0012901.s001.tif]

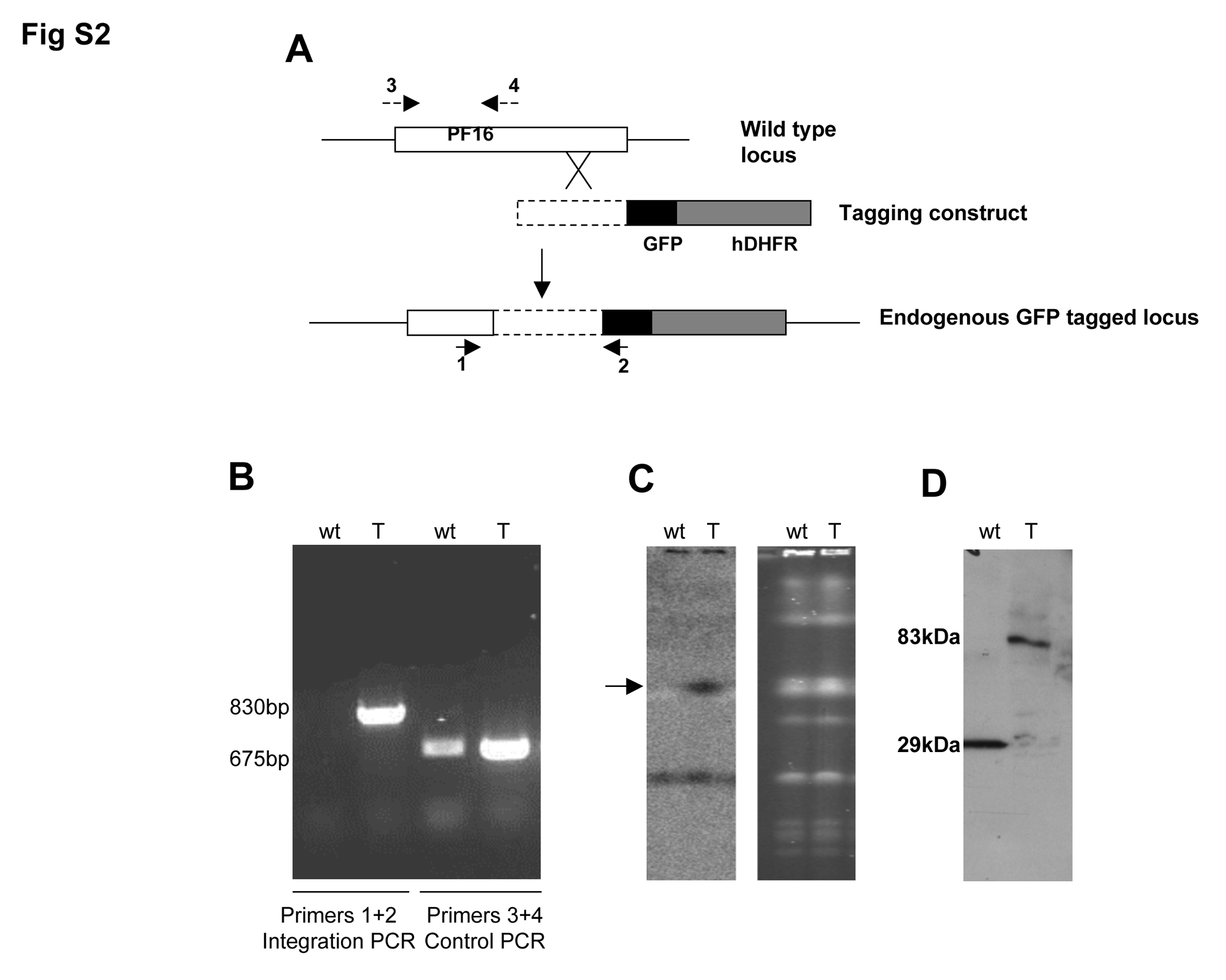

Supplement: Figure S2 — GFP Tagging of endogenous PF16 in P. berghei. A. Schematic representation of gene tagging construct used for endogenous GFP tagging by single homologous recombination. Position of primers 1–4 is (used for diagnostic PCR) is indicated B. Diagnostic PCR for integration of the transgenic construct for generating endogenous PF16-GFP parasites. Primer 1/2 shows integration across the region and 3/4 detects the template control. W represents the wild type and transgenic PF16-GFP is represented by (T) C. Correct integration of the transgenic construct (T) at chromosome 10 as described above, compared to the wild type locus (W). D. Western blot analyses using an anti-GFP antibody on control wild type gametocytes/gametes ubiquitously expressing soluble GFP (W) and transgenic male PF16-GFP-expressing gametes (T). (0.47 MB TIF) [file pone.0012901.s002.tif]

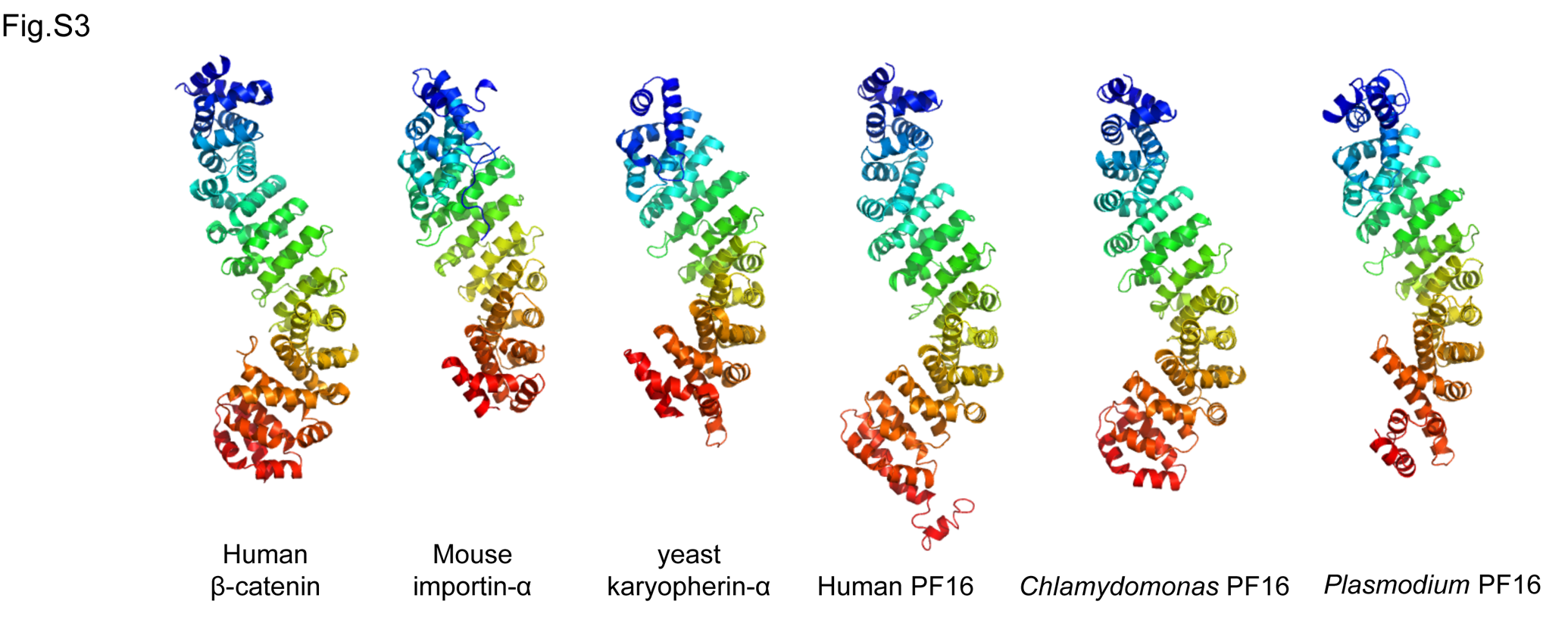

Supplement: Figure S3 — Structural Models of PF16. Comparison of the modelled Plasmodium, human and Chlamydomonas PF16 proteins with experimentally determined structures demonstrating the conserved nature of the ARM repeat. Structures shown are human β-catenin [PDB:1JDH], mouse importin α [PDB:1IAL] and yeast karyopherin α [PDB:1EE4]. All structures are shown in cartoon representation, coloured by spectrum from the N-terminus (blue) to C-terminus (red). (0.97 MB TIF) [file pone.0012901.s003.tif]
