# Supplementary material for: The Armadillo Repeat Protein PF16 Is Essential for Flagellar Structure and Function in Plasmodium Male Gametes
Source: PLoS One. 2010 Sep 23;5(9):e12901. doi: 10.1371/journal.pone.0012901 (PMC2944832; doi:10.1371/journal.pone.0012901)
Supplement: Materials and Methods S1 — (0.06 MB DOC) [file pone.0012901.s006.doc]

**“Supporting Methods”**

**Ethics statement**

All animal work has passed an ethical review process and was approved by the United Kingdom Home Office. Work was carried out in accordance with the United Kingdom "Animals (Scientific Procedures) Act 1986" and in compliance with "European Directive 86/609/EEC" for the protection of animals used for experimental purposes.

**Generation of transgenic parasites.**

***(a) Knockout construct***

Experiments were performed on *P. berghei* ANKA strain 2.34 parasites according to a described protocol [1] The *pf16* knock-out vector was constructed for double crossover homologous recombination in plasmid pBS-DHFR, which contains a *T. gondii dhfr/ts* cassette conferring resistance to pyrimethamine as described in our earlier studies[2,3] The knock-out construct was generated by inserting 0.48 kb of *pf16* 5’ UTR region upstream and 0.733kb of *pf16* 3’UTR region downstream of the *dhfr* cassette (sequences of primers used to amplify fragments from *P. berghei* genomic DNA are given below). The final knock-out construct was digested with *ApaI* and *XbaI* to release the fragment for transfection.

CCCCGGGCCCGCTCATTATTTCGTAAAAACACAC, N0051

GGGGAAGCTTGCAATATATTAAATGTGGTAATACATC, N0052

CCCCGAATTCCCATCCTATAAAAAATCATTTATTAAG, N0053

GGGGTCTAGACCGATACATGTTATGCATAGTG, N0054

***(b) GFP tagging construct***

To generate a PF16-GFP construct for single crossover homologous recombination, a 712bp region coding for the C-terminus of PF16 without the stop codon was inserted in frame and upstream of the *GFP* sequence in the plasmid p277 containing the human *DHFR* cassette and conveying resistance to pyrimethamine [2] Primers used to amplify this fragment are given below. Prior to transfection, the final construct was digested with BglII that cuts the plasmid in the middle of the insert, which is optimal for the homologous recombination event.

Transfected *P. berghei* parasites were selected with pyrimethamine pressure in drinking water (7mg/ml in DMSO dissolved in 500ml water) or by intra-peritoneal injection(250ug/100ul/mice in DMSO) according to a described protocol [1]. Transfection of *P. berghei* parasites with the knock-out construct was carried out a minimum of three times.

CCCCGGTACCGCATGCAACTGTTTATCACAAATAGC, T0021

CCCCGGGCCCTTTTACTTGTAAATATTTTAAGCATCCTGAC, T0022

***Genotype analysis of pf16 mutant parasites.***

(a) PCR analysis- two diagnostic PCR reactions were performed as illustrated in Fig.S1. In one reaction primer 1 and 2 were used to verify the correct integration of selectable marker at the *PF16* locus. The other set of primers, 3 and 4, was used to determine the deletion of the gene. The integration primer 1 is 5’ GGGTTATATAATAGGAATTCTTATTAAAAG 3’, INT N5 and primer 2 is 5’ GATGTGTTATGTGATTAATTCATACAC 3’. For set 3 and 4 the primers used were GTGCATATACACTAAAATGCTTGGC,N5KO1and GAGGTAAAGCACCTGCACGTAC, N5 KO2.

(b) Pulsed field gel electrophoresis (PFGE)-chromosomes of wild-type and *pf16* clones were separated on an LKB 2015 Pulsaphor system using a linear ramp of 60–500 s for 72 hr at 4V/cm. The gel was blotted and nylon membrane was hybridized with a probe that binds to the 3’UTR of DHFR/TS detecting both the endogenous *dhfr* locus (chromosome 7) and the modified *pf16* locus (chromosome 10).

(c) Southern analysis- genomic DNA from wild-type and mutant parasites was digested with *Eco* RI. The fragments were separated on a 0.8% agarose gel, blotted onto a nylon membrane, and probed with the fragment of the *PF16* 3’UTR used in the knock-out vector (see above).

For GFP-tagged transformants a similar analysis was followed, except the integration primer used was CTTTAAATATGTTATCTGAACTATGTAAAC, INTT2.

**Immunoblotting.**

Gametocytes were purified from the parasite blood using 48% nycodenz and protein samples were heated under reducing conditions (62.5 mM Tris-HCl, 2% w/v SDS, 10% glycerol, 50 mM DTT, 0.01% w/v bromophenol blue) for 10 minutes at 98°C. Proteins were separated on reducing 12.5 % SDS-PAGE gels and transferred onto Polyvinylidene Fluoride membranes (Amersham, UK). The blot was then incubated with a rabbit anti-GFP primary antibody 1:200 dilution (Molecular probes, UK) in TBS-Tween with 0.1% Tween concentration, for 45 minutes at RT or overnight at 4°C and subsequently incubated with secondary HRP-conjugated anti-rabbit IgG antibody for 30 minutes. The blot was developed using enhanced chemiluminescence (Amersham, UK).

**Complementation construct used in *Chlamydomonas*.**

In initial rescue attempts, we used a plasmid that includes the *P. berghei* coding sequence, as well as 228 nucleotides upstream of the translational start site and 673 nucleotides downstream of the translational stop site, cloned into the pGEM vector. In subsequent experiments, we generated a construct that included the *P. berghei* coding sequence as well as the *Chlamydomonas* 5’ and 3’ untranslated regions (UTRs) cloned into the pUC119 vector. The *Chlamydomonas* UTRs were obtained by PCR from the pB6D2 plasmid which rescues motility in the *pf16C,arg-* strain [4]. The 5’ UTR includes 1140 nucleotides upstream of the *Chlamydomonas* translational start site; the 3’UTR includes 1017 nucleotides downstream of the translational stop site. The resulting construct was sequenced to verify proper orientation of the UTRs relative to the *P. berghei* coding sequence. For all *Chlamydomonas* transformation experiments, high efficiency transformation was carried using the glass bead protocol [5] and 1µg of each plasmid. The transformants were plated on TAP[6] agar plates without arginine supplement. Successful transformants were picked into 96 well dishes containing TAP liquid media and scored for motility using a stereomicroscope.

**Accession numbers of PF16 protein from 33 species used to construct the tree as described in Figure 4A**

Mouse SPAG6 (blue), species name *Mus musculus* (reference NP_056588.1); Rat SPAG6, *Rattus norvegicus* (NP_001030132.1); Human SPAG6, *Homo sapiens* (NP_036575.1); Chicken, *Gallus gallus* (XP_418607.2); Frog, *Xenopus tropicalis* (NP_001015816.1); Zebrafish, *Danio rerio* (NP_001002210.1); Sea urchin, *Strongylocentrotus purpureum* (XP_794211.1); Sea anemone, *Nematostella vectensis* (XP_001634291.1); Choanoflagellate, *Monosiga brevicollis* (XP_001747810.1), Fungus, *Batrachochytrium dendrobatidis* (BDEG_01859.1); *Trypanosoma cruzi* (XP_807437.1); *Trypanosoma* brucei (XP_807437.1); *Leishmania infantum* (XP_001465274.1); *Leishmania major* (XP_001682894.1); *Giardia intestinalis* (XP_001705527.1); *Trichomonas vaginalis* (XP_001316961.1, XP_001583486.1, XP_001580482.1); *Naegleria gruberi* (XP_002681854.1); *Plasmodium berghei* PF16 (red), (XP_675975.1); *Plasmodium yoelii* (XP_728888.1); *Plasmodium falciparum* (XP_001347989.2); *Plasmodium vivax* (XP_001615422.1); *Toxoplasma gondii* (XP_002371202.1); *Perkinsus marinus* (EER08630.1); *Paramecium tetraurelia* (XP_001435920.1); *Tetrahymena thermophila* (XP_001009722.1|); Brown alga, *Aureococcus anophagefferens* (Protein ID:55534 Location: scaffold_60:7580-9289); *Phytophthora infestans* (EEY60977.1);Spikemoss, *Selaginella moellendorffii* (Protein ID:416565 Location: scaffold_31:621775-624018, Protein ID:99379 Location: scaffold_21:1160571-1162800); Moss, *Physcomitrella patens* (XP_001763837.1);*Micromonas pusilla* (EEH54980.1); *Micromonas RCC299* (XP_002506654.1);*Volvox carteri* (Protein ID:109629 Location:scaffold_7:369599-374444); *Chlamydomonas* PF16 (green), *Chlamydomonas reinhardtii* (GenBank U40057).

**Homology modelling as described in Figure. 4B and Supplementary Figure. S3**

I-TASSER was followed that undertakes threading, utilising profile-profile alignment, followed by structure assembly simulation and refinement [7]

Templates identified in this process for all three target sequences were human β-catenin [PDB:1JDH], yeast karyopherin α [PDB:1EE4/1WA5]. Additional templates for *Plasmodium* PF16 were zebrafish β-catenin [PDB:2Z6G], human importin-α [PDB:2JDQ] and mouse importin-α [PDB:1IAL] and for *Chlamydomonas* PF16 were zebrafish β-catenin [PDB:2Z6G] and mouse β-catenin [PDB:1I7W]. The global model quality was assessed using QMEAN, a composite scoring function [8]. The clustering-based scoring function QMEANclust was utilised to rank the models output from I-TASSER, and for each target sequence the top-ranking model was utilised for further analysis. RMSD deviations were calculated using SUPERPOSE [9].
