# Supplementary material for: The Armadillo Repeat Protein PF16 Is Essential for Flagellar Structure and Function in Plasmodium Male Gametes
Source: PLoS One. 2010 Sep 23;5(9):e12901. doi: 10.1371/journal.pone.0012901 (PMC2944832; doi:10.1371/journal.pone.0012901)
Supplement: References S1 — (0.02 MB DOC) [file pone.0012901.s007.doc]

**“Supporting References ”**

1. Janse CJ, Franke-Fayard B, Mair GR, Ramesar J, Thiel C, et al. (2006) High efficiency transfection of Plasmodium berghei facilitates novel selection procedures. Mol Biochem Parasitol 145: 60-70.

2. Liu Y, Tewari R, Ning J, Blagborough AM, Garbom S, et al. (2008) The conserved

plant sterility gene HAP2 functions after attachment of fusogenic membranes in

Chlamydomonas and Plasmodium gametes. Genes Dev 22: 1051-1068.

3. Tewari R, Dorin D, Moon R, Doerig C, Billker O (2005) An atypical mitogen-

activated protein kinase controls cytokinesis and flagellar motility during male gamete

formation in a malaria parasite. Mol Microbiol 58: 1253-1263.

4. Smith EF, Lefebvre PA (1996) PF16 encodes a protein with armadillo repeats and

localizes to a single microtubule of the central apparatus in Chlamydomonas flagella.

J Cell Biol 132: 359-370.

5. Kindle KL (1990) High-frequency nuclear transformation of Chlamydomonas

reinhardtii. Proc Natl Acad Sci U S A 87: 1228-1232.

6. Gorman DS, Levine RP (1965) Cytochrome f and plastocyanin: their sequence in

the photosynthetic electron transport chain of Chlamydomonas reinhardi. Proc Natl

Acad Sci U S A 54: 1665-1669.

7. Roy A, Kucukural A, Zhang Y (2010) I-TASSER: a unified platform for automated

protein structure and function prediction. Nat Protoc 5: 725-738.

8. Benkert P, Kunzli M, Schwede T (2009) QMEAN server for protein model quality

estimation. Nucleic Acids Res 37: W510-514.

9. Krissinel E, Henrick K (2004) Secondary-structure matching (SSM), a new tool for

fast protein structure alignment in three dimensions. Acta Crystallogr D Biol

Crystallogr 60: 2256-2268.
